# A statistical approach to selecting and confirming validation targets in -omics experiments

**DOI:** 10.1186/1471-2105-13-150

**Published:** 2012-06-27

**Authors:** Jeffrey T Leek, Margaret A Taub, Jason L Rasgon

**Affiliations:** 1 Department of Biostatistics, Johns Hopkins Bloomberg School of Public Health, 615 North Wolfe Street, Baltimore, MD 21205-2179, USA; 2 Department of Entomology, The Center for Infectious Disease Dynamics, and the Huck Institutes of Life Sciences, University Park, PA 16802, USA

## Abstract

**Background:**

Genomic technologies are, by their very nature, designed for hypothesis generation. In some cases, the hypotheses that are generated require that genome scientists confirm findings about specific genes or proteins. But one major advantage of high-throughput technology is that global genetic, genomic, transcriptomic, and proteomic behaviors can be observed. Manual confirmation of every statistically significant genomic result is prohibitively expensive. This has led researchers in genomics to adopt the strategy of confirming only a handful of the most statistically significant results, a small subset chosen for biological interest, or a small random subset. But there is no standard approach for selecting and quantitatively evaluating validation targets.

**Results:**

Here we present a new statistical method and approach for statistically validating lists of significant results based on confirming only a small random sample. We apply our statistical method to show that the usual practice of confirming only the most statistically significant results does not statistically validate result lists. We analyze an extensively validated RNA-sequencing experiment to show that confirming a random subset can statistically validate entire lists of significant results. Finally, we analyze multiple publicly available microarray experiments to show that statistically validating random samples can both (i) provide evidence to confirm long gene lists and (ii) save thousands of dollars and hundreds of hours of labor over manual validation of each significant result.

**Conclusions:**

For high-throughput -omics studies, statistical validation is a cost-effective and statistically valid approach to confirming lists of significant results.

## Background

**Figure 1:**
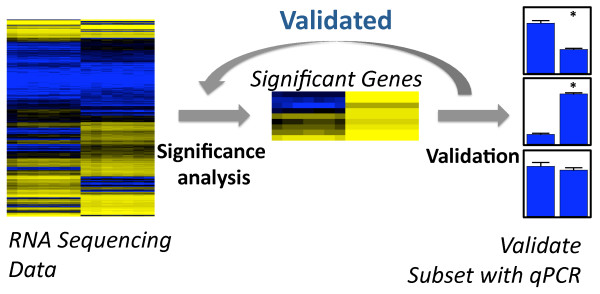
**Validation strategy schematic.** A set of RNA sequencing data is analyzed using statistical models (blue = high expression, yellow = low expression) and a list of significant genes is identified at a fixed false discovery rate (FDR). From the list of significant genes a few - usually the most statistically significant - are validated with the independent validation technology quantitative PCR (qPCR). Ideally the confirmation with inde-pendent technology can be used to validate the entire list of significant genes.

High-throughput molecular biology experiments are now commonplace. Technologies such as microarrays [1] and next-generation sequencing [2] are routinely used to measure thousands or millions of variables for each sample in a study. From the measured variables, several hundred may be “statistically significant” in a typical experiment [3]. Usually a much smaller number are manually validated, typically those with the most significant p-values, using an independent validation technology (Figure 1). One goal of manual validation is to confirm specific biological findings. For example, it may be of interest to confirm a specific SNP is associated with a complex phenotype or to confirm a specific protein-protein interaction. It has been pointed out that confirming only a small number of results with an independent technology is often less convincing than the original experiment [4].

But the true advantage of genomic technologies lies in their ability to generate global hypotheses about the genome, epigenome, transcriptome, or proteome. Network analyses, systems biology, and gene set analysis fall into this category, since they produce results that relate many features simultaneously. Manual validation is also commonly used to support these global hypotheses by: (i) providing evidence for the technology and methods that generate a set of significant results [5,6] or (ii) ensuring that downstream functional analyses based on sets of significant features - such as gene set analysis [7] - are based on accurate lists of features.

When the goal of validation is to confirm only specific biological conclusions, investigators may choose the most biologically interesting features to subject to manually validate. However, when the goal is either to implicitly or explicitly validate methods or lists of significant results there is no standard approach for validation, so researchers do one of the following: (i) manually validate only the set of top hits - based on statistical or biological significance, (ii) to manually validate the entire set of significant results, or (iii) validate a small random subset of results. Since manually confirming results is costly and time-consuming, strategy (i) or (iii) are more common.

When the goal is to support a statistical method or list of features, analyzing only the most significant hits may not be sufficient [4]. This type of validation is statistically unsound for validating lists of genes, since using a strongly biased sample will lead to strongly biased statistical analyses. If there are more false positives than expected, due to batch effects or other artifacts [8,9], the broader scientific conclusions of the study may be strongly biased. For example, gene set enrichment analysis may give inaccurate results when correlation is not accounted for appropriately [10]. Furthermore, the choice of results to subject to manual confirmation is often based on biological intuition, which is subject to human biases and subjective error [11].

Here we suggest that the third approach, statistical validation of significant results, is the most effective. We present a new statistical procedure for calculating the probability that a result has been validated. These probabilities are calculated based on manually confirming a small random sample of significant results. This new statistical method estimates the accuracy of lists of features, or the quality of technology and statistical methods, and hence the strength of global genomic hypotheses. Furthermore, statistical validation is cheaper and less time consuming than confirming every significant result.

Statistical methods have been developed for calculating internal validation within studies [12] and for optimizing the number of confirmatory experiments to perform given a fixed set of costs [13]. But, to our knowledge, this is the first statistical method for evaluating independent experimental validation based on a subsample of significant results.

## Methods

### A statistical approach to validation

Interesting results in high-dimensional studies are the assays that are statistically significant at a specified false discovery rate (FDR). The FDR can be thought of as the acceptable level of false positives among a set of significant results [3]. If 100 variables are significant at an FDR ≤ 5*%*, then we expect no more than 0.05 × 100 = 5 false discoveries. Applying an independent technology to confirm all 100 variables and finding 5 or fewer false positives would verify this claim. However, applying independent technologies or functional assays to individual discoveries is often costly and time consuming.

Here we propose to experimentally test a random sample of significant results with an independent technology and confirm the false discovery rate with a statistical procedure. The approach consists of manually confirming a sample of *n* hits with the independent technology, determining the number of false positives, *n*_*FP*_, and calculating the probability the true proportion of false positives, *Π*_0_, is less than the claimed FDR of $\hat{\alpha }$. When a subset of features is claimed to be significant at a specified FDR, *α*, then the expected proportion of false positives among the significant results is *α*. The expected proportion of false positives in the validation sample *Π*_0_, should then be approximately equivalent to the original FDR. We can then use the expected proportion of false positives in the validation sample to confirm the original FDR estimate.

If the probability $\mathrm{Pr}(\Pi_{0}\leq \hat{\alpha }|n_{\mathrm{FP}},n)$ is larger than 0.5, then the validation sub-study supports the original FDR estimate, although larger values are required to strongly support validation. Using the posterior distribution, it is also possible to calculate a posterior credible interval for the false discovery rate, *Π*_0_, as a measure of variability. This probability represents a direct measurement of concordance, unlike statistics like the correlation between the original and validation statistics which measure only agreement and depend on the scale of the measurements being taken [14].

### Calculating validation probabilities

Suppose that there are *m* significant hits at an FDR of *Π*_0_, and *n* of them are sampled randomly for validation. For each of the *n* probes let *δ*_*i*_ = 1 if probe *i* is a false positive according to the independent technology and *δ*_*i*_ = 0 if not. Each probe may have a different probability of being a false positive, so Pr(*δ*_*i*_ = 1|*p*_*i*_) = *p*_*i*_ where *p*_*i*_ is drawn from a distribution, *f*(*p*) such that E_*f*(*p*)_[*p*] = *Π*_0_. The distribution of *δ*_*i*_ can be written as:

(1) $$\begin{array}{lll} f(\delta_{i})\; & =\;\overset{1}{\underset{0}{\int }}p^{\delta_{i}}{\left(1-p\right)}^{1-\delta_{i}}\times f(p)\mathrm{dp}\; & \;\\ \; & =\;\left\{\begin{array}{c} \overset{1}{\underset{0}{\int }}p\times f(p)\mathrm{dp}=\Pi_{0}\;\mathrm{if}\;\delta_{i}=1\\ \overset{1}{\underset{0}{\int }}(1-p)\times f(p)\mathrm{dp}=1-\Pi_{0}\;\mathrm{if}\;\delta_{i}=0 \end{array}\right)\; & \;\\ \; & =\;\Pi_{0}^{\delta_{i}}{\left(1-\Pi_{0}\right)}^{1-\delta_{i}}\; \end{array}$$

So the number of false positives, *n*_*FP*_ has a binomial distribution with parameter *Π*_0_. We assume a *Beta*(*ab*) conjugate prior distribution for *Π*_0_[15], so the posterior distribution is a *Beta*(*a* + *n*_*FP*_*b* + *n*−*n*_*FP*_) [16]. This result relies on the independence of the validation experiments, but does not rely on the rate of true or false positives. The potential reasons for dependence between validation tests include batch and other technical artifacts [9]. However, methods have been developed to address these potential sources of dependence [8,17], which lead to nearly independent hypotheses [18].

Using this posterior distribution it is straightforward to calculate the probability that the FDR (*Π*_0_) is less than the claimed level $\left(\hat{\alpha }\right)$: $\mathrm{Pr}(\Pi_{0}<\hat{\alpha }|n_{\mathrm{FP}},n)$. We can also calculate the posterior expected value of the FDR using the mean of the posterior, $\frac{a+n_{\mathrm{FP}}}{a+b+n}$, which can give us an idea of the actual FDR of our original results. For our analysis, we made the assumption that the prior distribution for *Π*_0_ was *U*(0,1), by setting the parameters to the Beta function to *a* = *b* = 1. This prior is somewhat conservative, since it is likely that many of the results in the validation experiment will be true positives.

In some cases it may be useful to encode the belief that most of the results will be true positives in the prior by choosing values of *a* and *b* that put greater prior weight on higher validation probabilities. Specifically, a much less conservative prior choice would set the mean of the prior distribution to be the observed FDR for the validation targets in the original study $\hat{\alpha }=\frac{a}{a+b}$. Since this choice permits a range of solutions for *a* and *b*, one could select the choice that maximizes the prior variance $\frac{\mathrm{ab}}{{\left(a+b\right)}^{2}(a+b+1)}$. This could be accomplished by choosing *a* to be a small value like 0.01 and setting $b=\frac{1-\hat{\alpha }}{\hat{\alpha }}\times a$.

For small values of $\hat{\alpha }$ this prior may influence the prior probabilities and make it difficult to compare validation at different FDR levels. This is a particular concern given the known variability in FDR estimates, particularly for small sample sizes or low FDR levels [19]. In general, conservative and non-adaptive prior distributions will lead to less potential for bias and greater comparability across FDR levels. Our R functions for calculating the validation probabilities allow for different choices of *a* and *b*. However, the validation probability is somewhat robust to prior choice (Results).

### Bootstrap confidence intervals for the validation probabilities

It may be of interest to determine the variability of the validation probability. One potential approach is to calculate a bootstrap confidence interval for the posterior probability [20]. The basic approach is as follows. 

1. For *b* = 1,…,*B*bootstrap samples, take a random sample of size *n* with replacement form the *n* validation results. Calculate the number of false positives and calculate the null statistic: ${\mathrm{Pr}}^{b}(\Pi_{0}<\hat{\alpha }|n_{\mathrm{FP}}^{b},n)$.

2. Calculate the 2.5th and 97.5th quantiles of the distribution of null statistics, ${\mathrm{Pr}}^{b}(\Pi_{0}<\hat{\alpha }|n_{\mathrm{FP}}^{b},n)$. Use these values as a 95% confidence interval for the validation probability.

The bootstrap is not justified for small sample sizes, and when the validation sample size is small, these bootstrap confidence intervals may not have the appropriate coverage.

### Choosing the FDR level and sample size

An important question for statistical validation is: How does one choose the FDR level and the validation sample size to use? To answer this question, suppose that in a given study for each FDR cutoff *q*, there are *n*_*sig*_(*q*) significant genes. The goal is to find the minimum number of sampled results required to achieve a high validation probability Pr(*Π*_0_ <*q*|*n*_*FP*_,*n*), for the case where the results would be confirmed with a perfect independent technology. The minimum validation sample size for FDR cutoff *q* can be found by solving the following optimization problem:

(2) $$\underset{n}{\min}\mathrm{Pr}(\Pi_{0}<q|n\times q,n)>\text{Target Probability}$$

In other words, what is the minimum validation sample size needed to get at least the target validation probability, assuming that *q* × *n* false positives will be observed?

Here, as in any sample size calculation, we must estimate the effect size - in this case the expected number of false positives in the validation set. In our examples, we estimate the effect size as the observed FDR for the validation targets. However, our R functions allow for alternative choices of the expected FDR for each true FDR level. If a user chooses higher FDR levels than the observed values, the minimum sample size will be smaller to confirm that higher FDR threshold.

This optimization problem can be solved for any specific study based only on the set of p-values for the original analysis performed. For a fixed target probability and a fixed false discovery rate threshold, the minimum sample size will be fixed as long as the number of significant features *n*_*sig*_(*q*) is sufficiently large. The reason is that the optimization is over only the single variable *n*, when *q* and the Target Probability are fixed.

**Figure 2:**
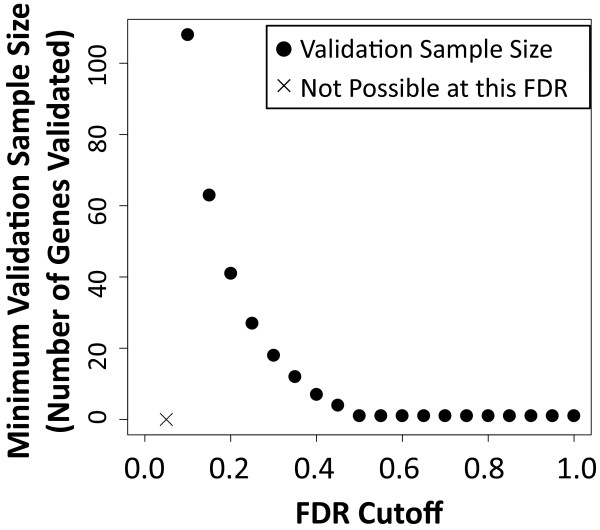
**Minimum Validation Sample Size Versus FDR Cutoff.** A plot of the minimum validation sample size required using sampling to achieve a target validation probability of 0.5, assuming that the experimental technology, statistical method, and validation technology are accurate. This plot is based on the results of a specific study and can be used to plan validation experiments. A ∙ indicates the minimum sample size for a fixed FDR cutoff and a × indicates that for that FDR threshold the target validation probability can not be achieved.

As an example of this procedure, we use the data from the first simulated study (of 100) in the errorless validation simulation as described in the Results. Based on the p-values from that study, we calculated the minimum validation sample size needed for each FDR threshold to achieve a target validation probability of Pr(*Π*_0_ <*q*|*n* × *q*,*n*) ≥ 0.5 (Figure 2). For an FDR of 5%, it is not possible to achieve the desired validation probability. For increasing FDR cutoffs, the required validation sample size decreases. This is not surprising, since we have shown in the previous section that validation is more likely at higher FDR thresholds. The lower the FDR threshold used for validation, the more convincing the validation may be, so the investigator can calculate this curve for any given study and use the results to decide how many results to validate based on available resources.

If the authors have designed their study using our estimates of the minimum validation sample size, and the validation probability is low, then it is likely that *Π*_0_ is greater than the claimed FDR level. If however, they choose to validate many fewer targets than suggested by the minimum validation sample size, it is ambiguous whether the sample size was too small or the FDR did not validate.

### Calculating qPCR validation costs

Manually confirming genomic results with independent technologies or functional assays can be costly and time consuming, since most validation technologies must be performed one gene, transcript, or protein at a time. There are a large number of validation technologies, but one of the most commonly used is quantitative PCR (qPCR). To compare costs associated with different strategies we use qPCR validation for gene expression studies as an example. The results presented here are representative of the results for any costly independent confirmation experiment.

We estimated the costs associated with two different qPCR technologies: SYBrGreen and TaqMan. For TaqMan we assumed that three genes and a reference gene were multiplexed in each reaction, which is theoretically possible but optimistic in practice. SYBrGreen reactions also included a reference gene, but were not assumed to be multiplexed. We calculated costs as follows: $250 for each TaqMan probe, $150 for Mastermix for each plate for both SYBrGreen and TaqMan, and $4 for each 96-well plate. We assumed each reaction was replicated three times to ensure accurate measurements - a typical approach taken in validation experiments. We also made the assumption that one research assistant, working full time and paid $40,000 per year, could run and analyze four 96-well plates per day. For the purposes of our analysis we assumed 22 working days per month.

Based on these assumptions, we can calculate the cost and time required for validating *n*_*genes*_ genes on *n*_*samples*_ samples for each technology. For TaqMan, after accounting for multiplexing the number of plates run is $\frac{n_{\mathrm{genes}}\times n_{\mathrm{samples}}}{96}$, so the cost (*C*_*TaqMan*_) and time (*T*_*TaqMan*_) for the validation experiment are as follows.

(3) $$\begin{array}{lll} C_{\mathrm{TaqMan}}\; & =\;\underset{\text{Primer Costs}}{\underset{︸}{\$250\times n_{\mathrm{genes}}}}\;\\ \; & +\;\underset{\text{Reagent Costs}}{\underset{︸}{\left(\$4+\$150)\times \lfloor \frac{n_{\mathrm{genes}}\times n_{\mathrm{samples}}}{96}\right\rfloor }}\; & \;\\ \; & +\;\underset{\text{Personnel Costs}}{\underset{︸}{\$40,000\times T_{\mathrm{TaqMan}}}}\; & \; \end{array}$$

(4) $$\begin{array}{ll} T_{\mathrm{TaqMan}}\; & =\;\frac{1}{4\times 22\times 12}\times \lfloor \frac{n_{\mathrm{genes}}\times n_{\mathrm{samples}}}{96}\rfloor \; \end{array}$$

For the SYBrGreen validation, reactions can not be multiplexed. However, these reactions also do not incur the primer costs of the TaqMan reactions. So the cost (*C*_*SG*_) and time (*T*_*SG*_) for the validation experiment are as follows.

(5) $$\begin{array}{lll} C_{\mathrm{SG}}\; & =\;\underset{\text{Reagent Costs}}{\underset{︸}{\left(\$4+\$150)\times \lfloor \frac{n_{\mathrm{genes}}\times n_{\mathrm{samples}}\times 3\times 2}{96}\right\rfloor }}\;\\ \; & +\;\underset{\text{Personnel Costs}}{\underset{︸}{\$40,000\times T_{\mathrm{SG}}}}\; & \; \end{array}$$

(6) $$\begin{array}{ll} T_{\mathrm{SG}}\; & =\;\frac{1}{4\times 22\times 12}\times \lfloor \frac{n_{\mathrm{genes}}\times n_{\mathrm{samples}}\times 3\times 2}{96}\rfloor \; \end{array}$$

In these equations the terms inside the floor operators ⌊·⌋ represent the number of plates needed to run the reactions, which must be multiplied by the fixed costs for those plates. From these equations, it can be seen that manual confirmation of gene expression results using either Taqman or SYBrGreen is costly and time consuming. Taqman is slightly more expensive, but slightly less time consuming.

## Results and Discussion

### Non-random validation can not be used to confirm a complete list of significant results

Manually confirming only the most significant results (Figure 1) is probably the most common validation strategy in genomic studies. To highlight the need for a statistical approach to validation, we consider two representative studies that use this strategy. In both cases, the implicit assumption was made that validating the most significant results was sufficient to support the accuracy of a statistical method or an entire list of significant results [21,22]. This assumption is often made by investigators who perform global gene-set enrichment or functional analysis of significant features, after manually confirming only the most extreme results. This has led some to suggest that individual validation experiments do not reflect the quality of genomic results [4].

In the first study 388 cassette exons were identified with a Bayesian network as Nova alternative splicing targets with FDR < 1%. A convenience sample of 31 exons was validated, yielding 28 true positives [22]. We calculate the posterior distribution for the estimated FDR among the validated results using the Beta distribution derived from equation 1 (see Methods). Based on this posterior distribution we obtained a 95% posterior credible interval for the FDR of (0.04, 0.25). Our results suggest that based on the validation sample the true FDR for the original analysis is likely between 4% and 25%, substantially higher than the reported FDR. This is not surprising, since the expected number of false discoveries at an FDR of 1% is 0.01×31=0.31. Since the claimed FDR does not match the validation results, the probability of validation (see Methods) is low, $\mathrm{Pr}(\Pi_{0}\leq 0.01|n_{\mathrm{FP}}=3,n=31)=2.87\times 10^{-4}$.

In the second study 53 master regulator transcription factors were identified for a “mesenchymal” gene expression signature at an FDR of 5% [21]. The two most significant master regulators were confirmed functionally. The investigators did not report the FDR for the hits that were functionally confirmed, so it is not possible to directly calculate the validation probability. However, we make the conservative assumption that these hits were also significant at an FDR of 5%, although the FDR is likely much lower for the top two hits. Using the less stringent threshold of 5% the 95% credible interval for the true FDR is wide (0.01, 0.70). The reason is that although 100% of tested results were confirmed using the functional test, the sample size is too small to confirm the original false discovery rate claims. Thus, the corresponding probability of validation Pr(*Π*_0_ ≤ 0.05|*n*_*FP*_ = 0,*n* = 2) = 0.14 is low.

In these studies, only a small fraction of the top hits were confirmed with independent technology. However, the entire list of significant results was used in each case to form a biological picture and interpretation of the results. The corresponding validation probabilities suggest that this confidence in the entire set of significant results is not sufficiently justified. These examples are illustrative of typical validation strategies and suggest the need for a new approach for supporting lists of significant results with independent measurements. In the next section, we show that manually confirming a random sample of results is more effective than manually confirming only the most significant results when the goal is to provide statistical support for validation.

### Statistical validation can be used to confirm lists of significant results

To compare: (i) manually confirming only the most significant results and (ii) statistical validation, an example is needed where every single genomic feature is assayed on both the original technology and an independent validation technology. Such data sets are rare, since most measurements from high-throughput -omics studies are not confirmed with independent technology because of time or financial constraints.

To make this comparison, we obtained expression data for 805 genes from a study of brain and reference tissue measured by both RNA sequencing and an independent technology, quantitative PCR (qPCR) [23]. These data can be thought of as an experiment where every gene has been subjected to independent confirmation. Previous studies have shown that the qPCR and RNA-sequencing approaches produce comparable results in this experiment, suggesting that the validation probability should be high [23]. We use RNA-sequencing and quantitative PCR (qPCR) as a representative example of the type of independent manual confirmation that is performed in genomic experiments. However, our results easily generalize to any costly/time-consuming independent validation, whether it is with an independent technology or using a functional assay.

We compared the two strategies: (i) statistical validation by manual confirmation of a random sample of 20 genes significant at an FDR of 10% and (ii) manual confirmation of only the top 20 genes. We performed a likelihood ratio test to identify differentially expressed genes based on RNA-sequencing [23,24]. Genes with a qPCR fold-change above 0.25 were considered true positives. The estimated FDR for the top 20 genes was 1.09×10^−7^. All 20 were true positives according to qPCR. The FDR threshold is extremely low so a huge number of results would need to be confirmed to convincingly support the FDR claim. It is not surprising then, that the 95% posterior credible interval (0.001,0.161) does not cover the original FDR estimate and the validation probability for the 20 best hits is low $\mathrm{Pr}(\Pi_{0}\leq 1.09\times 10^{-7}|n_{\mathrm{FP}}=0,n=20)=2.28\times 10^{-6}$.

For a random sample of size 20 from among the 591 genes significant at an FDR of 10%, on average 1.65 were false positives. Since this is a random sample of results with FDR < 10%, the validation probability is calculated at the 10% threshold (Methods), yielding a 95% posterior credible interval (0.02, 0.28) that covers 10% and a substantially higher value of Pr(*Π*_0_ ≤ 0.10|*n*_*FP*_ = 1.65,*n* = 20) = 0.44.

These results suggest that choosing a random sample of results based on a higher FDR threshold made it easier to statistically support significance claims in genomic studies. In the next section, we show that when attempting to statistically validate a set of significant results, it is generally better to choose a higher FDR threshold.

### Statistical validation is more likely at higher FDR thresholds

**Figure 3:**
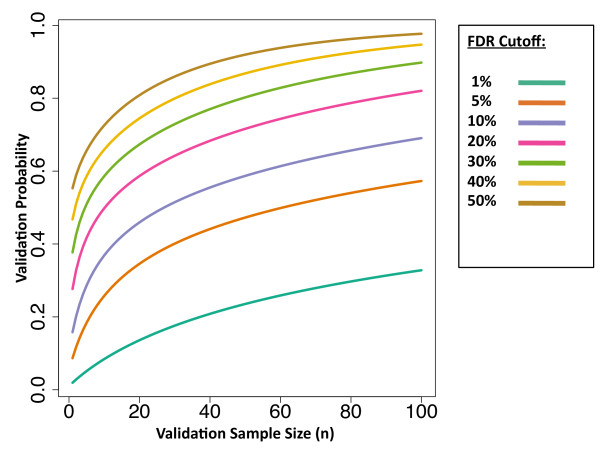
**Validation Probability by Sample Size.** A plot of the validation probability versus the sample size, for various FDR cutoffs assuming that (0.7 × FDR level × Validation sample size) false positives are observed in the validation set. For any sample size, the validation probability is higher when the FDR cutoff is larger.

As an example, suppose that in a given study the observed number of false positives is fixed at 0.7 × FDR level × the validation sample size, so the validation probability is expected to be high. In this scenario, the validation probability increases with increasing validation sample size (Figure 3). But for a fixed validation sample size higher FDR thresholds on the original data set lead to higher validation probabilities.

The reason is if only the most significant results are chosen for manual confirmation, then the claimed FDR for these results will be very small. Even if all of the results are confirmed as true positives, it is difficult to prove the FDR claims. For example, suppose the top 10 results correspond to an FDR of 1×10^−5^. This suggests that for every 100,000 results there should only be one false positive. Strong evidence for this claim would require confirming a huge number, hundreds of thousands, of results. Alternatively, if 10 results are validated at a higher FDR threshold like 0.50, then we expect about 5 false positives. If only 3 or 4 are observed, this would lend reasonable support to the FDR claims.

### Simulation study to evaluate the validation probability

The validation probability is a measure of how well the conclusions of the original study are supported by the validation sample. We have demonstrated the potential utility of this approach using real data sets.

We also consider three simulated scenarios to evaluate the properties of the validation probability parameter estimates. For each scenario, we calculate the validation probability using a conservative prior (*a* = * b * = 1) and the adaptive prior described in Methods (*a* = 0.01 and $b=\frac{1-\hat{\alpha }}{\hat{\alpha }}\times a$). In each case, we simulated 100 gene expression experiments with 1,000 genes, 300 of which were differentially expressed. In all cases, we assume that all genes which were significant at the given FDR cutoff were validated (exhaustive validation).

In the first case, we assume that the independent technology perfectly distinguishes the true positives from the false positives, so that *n*_*FP*_ is exactly the number of genes which were not in the set of 300, but which were significant at the given FDR cutoff in the original study. In the second case, we add an element of randomness to the validation results, so that a gene in the set of 300 may not be declared differentially expressed in the validation. In determining the validation results, the random outcome is based on a larger sample size than the original experiment but may give a different result. This simulation mimics a more realistic scenario where the validation technology is not perfect [25]. In both of these first two cases, the validation data should match the results of the original experiment, so the validation probabilities should be high, and the posterior expected value of the FDR based on the validation should be at or below the FDR from the original data set. In the third simulated case, we set the parameters so that the original technology is incorrect 1/3 of the time (Table 1). However, the independent technology correctly identifies the true differentially expressed genes, so that *n*_*FP*_ is again the number of genes not in the set of 300, but which were significant at the given FDR cutoff in the original data set.

**Table 1:** Simulation study to assess the properties of the validation probability

**Scenario**	**Quantity**	**FDR**	**FDR**	**FDR**
		**5%**	**10%**	**50%**
Errorless validation	Median Validation Probability	0.72	0.91	1.00
Prior = Uniform	Validation Probability IQR	(0.58, 0.87)	(0.72, 0.98)	(1.00,1.00)
	FDR 95% Credible Interval Coverage	0.98	0.68	0.00
	Median Posterior Expectation of FDR	0.04	0.07	0.35
Validation subject to error	Median Validation Probability	0.64	0.87	1.00
Prior = Uniform	Validation Probability IQR	(0.37, 0.83)	(0.71, 0.95)	(1.00,1.00)
	FDR 95% Credible Interval Coverage	0.98	0.68	0.00
	Median Posterior Expectation of FDR	0.05	0.08	0.24
Results should not validate	Median Validation Probability	0.00	0.00	0.25
Prior = Uniform	Validation Probability IQR	(0.00,0.00)	(0.00,0.00)	(0.15,0.51)
	FDR 95% Credible Interval Coverage	0.00	0.00	0.96
	Median Posterior Expectation of FDR	0.35	0.37	0.52
Errorless validation	Median Validation Probability	0.83	0.94	1.00
Prior = Adaptive	Validation Probability IQR	(0.70,0.94)	(0.79,0.99)	(1.00,1.00)
	FDR 95% Credible Interval Coverage	0.88	0.63	0.00
	Median Posterior Expectation of FDR	0.04	0.07	0.35
Validation subject to error	Median Validation Probability	0.76	0.91	1.00
Prior = Adaptive	Validation Probability IQR	(0.49, 0.91)	(0.78, 0.97)	(1.00,1.00)
	FDR 95% Credible Interval Coverage	0.91	0.77	0.00
	Median Posterior Expectation of FDR	0.04	0.07	0.23
Results should not validate	Median Validation Probability	0.00	0.00	0.25
Prior = Adaptive	Validation Probability IQR	(0.00,0.00)	(0.00,0.00)	(0.15,0.51)
	FDR 95% Credible Interval Coverage	0.00	0.00	0.95
	Median Posterior Expectation of FDR	0.35	0.37	0.52

For each of three scenarios and two choices for the prior distribution, 100 simulated gene expression studies were generated with 1,000 genes each. This table reports the median (25th percentile, 75th percentile) of the validation probability across the 100 studies, the coverage proportion of the 95% posterior credible interval for the estimated FDR in each scenario, and the median posterior expectation of the FDR.

When the original experiment is supported by the validation data and the validation test is perfect (Table 1 Scenario: Errorless validation) the validation probabilities are high. This is true even in the more realistic setting where the independent technology is subject to random error (Table 1, Scenario: Validation subject to error). The validation probability is also larger for larger values of the FDR, consistent with observations on the real examples described above. The posterior expected value of the FDR based on the validation data is at or below the FDR from the original data set, which is consistent with expectations. In the case where the validation experiment does not support the original experiment, the validation probabilities are much lower (Table 1, Scenario: Results should not validate). Here, we can see the posterior expected value of the FDR exceeds the FDR from the original data set, again as expected.

Interestingly, the coverage probabilities for the 95% posterior credible intervals are greater for lower values of the FDR. The reason is that the estimate of the FDR is conservatively biased and this bias is stronger for higher FDR cutoffs. The bias means that the posterior credible intervals cover the true FDR, but frequently do not contain the original FDR estimate because of the conservative bias.

The adaptive prior led to slightly higher validation probabilities but lower coverage of the 95% credible intervals - suggesting that the adaptive prior may be slightly anti-conservatively biased. However, the estimates were not wildly different suggesting relative robustness of the validation probabilities to the choice of prior.

### Statistical validation is cheaper and less time-consuming than manually confirming all significant results

An alternative strategy to confirming only the most significant results is to manually curate every significant result using an independent technology or assay. However, this approach is both costly and time consuming (see Methods). As an example of the potential advantages of the proposed statistical validation strategy, we analyzed the data from six gene expression microarray experiments (Table 2). For each experiment, we performed a standard significance analysis and identified the genes differentially expressed at a false discovery rate of 5% [3]. In many cases, a huge number of genes are identified as significantly differentially expressed. We used the equations (2-5) to calculate the costs associated with manually confirming all of the genes in the significance lists. These experiments would clearly never be performed, as the costs in terms of both money and time are prohibitive. For comparison purposes, we also calculated the costs performing statistical validation using the minimum sample size required to obtain an expected minimum validation probability of 0.5. Note that since the target probability (0.5) and the FDR level (5%) are fixed, the validation sample size is always the same.

**Table 2:** Statistical validation analysis of data from six microarray experiments obtained from GEO

**Study GSE #**	**# DE Genes**	**# Samples**	**Fraction of DE Genes**	**Cost**	**Cost**
			**Required for Statistical Validation**	**(Manual)**	**(Statistical)**
GSE10245	6,742	58	3.57%	3.85 years	0.14 years
				$2.5e6	$8.8e4
GSE11492	333	8	72.37%	0.03 years	0.02 years
				$8.9e4	$6.4e4
GSE17913	739	79	32.61%	0.58 years	0.18 years
				$3.0e5	$9.8e4
GSE16032	343	10	70.26%	0.03 years	0.02 years
				$9.3e4	$6.5e4
GSE16538	1,624	12	14.83%	0.19 years	0.03 years
				$9.3e4	$6.6e4
GSE11524	2,295	30	10.50%	0.68 years	0.07 years
				$7.1e5	$7.5e4

For each data set, differential expression was calculated with respect to the primary biological variable. Fomnr each experiment, the number of genes differentially expressed at 5% is reported. In each case, 241 genes are required for statistical validation, for each study we present the fraction of the DE genes required for statistical validation. Tthe cost in dollars and graduate student years of manually confirming the whole list of DE genes or only the DE genes needed for statistical validation is also reported.

When performing statistical validation, only a subsample of results must be confirmed, so the costs are substantially lower. Although the costs are still high in this case, they are substantially less prohibitive than manually confirming an entire list of results. The larger the list of significant hits, the more pronounced the savings from statistical validation. From the table, it is clear that validation at the FDR 5% level is both costly and time consuming, even using statistical validation. As we have shown, higher FDR thresholds lead to smaller minimum validation sample sizes (Figure 3). The results in Table 2 suggest that choosing a higher FDR threshold for statistical validation may be more economical.

## Conclusions

Genomic technologies are, by their very nature, designed for hypothesis generation. In some cases, the hypotheses that are generated require that genome scientists confirm findings about specific genes or proteins. But the true advantage of high-throughput technology is that global genetic, genomic, transcriptomic, and proteomic behaviors can be observed. Validating high-dimensional experimental results with independent technologies and assays is critical. Without independent validation, it is impossible to distinguish discoveries from spurious results due to technological artifacts, inappropriately applied statistical methods, or unmeasured latent variables.

Here we have introduced the first method for statistically quantifying the strength of a validation experiment. We have proposed a new statistical approach to validation that focuses on the last two cases. We have illustrated this approach with representative examples from the literature and an extensively validated RNA-sequencing experiment. We have also shown that statistical validation may be substantially more cost effective than manually confirming every significant result. Our work suggest that (i) the validity of lists of significant results can be inferred from confirming a small random sample of results, (ii) that this approach may reduce the costs to investigators, and (iii) statistical validation allows researchers to quantify the quality of their validation experiments. A web application for calculating validation probabilities is available at: http://www.biostat.jhsph.edu/∼jleek/validate/. R code for reproducing all the results and simulations in this paper is also available from that site.

## Competing Interests

The authors declare that they have no competing interests.

## Authors contributions

JL designed and conceived the study. JL and MT performed the statistical analysis. JR performed the analysis of validation costs for microarrays. JL, JR, and MT wrote the paper. All authors read and approved the final manuscript.
